# Chromosome 22q11.2 Microduplication Syndrome: A Review of the Literature and 12 New Cases

**DOI:** 10.3390/genes17070844

**Published:** 2026-07-22

**Authors:** Maria Bisba, Eirini Louizou, Spiros Vittas

**Affiliations:** 1 Department of Quality and Vigilance, Vigi-Care, 11525 Athens, Greece; mpismpamaria@gmail.com; 2 Department of Molecular Genetics, Cardea Medical, 15233 Athens, Greece; louizoueirini1978@gmail.com

**Keywords:** 22q11.2 microduplication syndrome, array-CGH, variable expressivity, incomplete penetrance, genetic counseling

## Abstract

Background/Objectives: 22q11.2 microduplication syndrome is a rare genetic disorder characterized by the presence of one or two additional copies of a segment within the 22q11.2 region of chromosome 22. While much of the literature has focused on the deletion variant leading to DiGeorge syndrome, the duplication counterpart has gained increasing attention due to its clinical variability and under-recognition. This review aims to deliver new possibilities to genetic counseling that can be provided in prenatal and postnatal cases as the phenotype of 22q11.2 microduplication carriers cannot be fully predicted. Methods: In the present study, a total of 12 (5 prenatal and 7 postnatal) cases were diagnosed through array-CGH and combined with 679 (95 prenatal and 584 postnatal) cases reported in the literature. This review summarizes the published evidence available up to April 2025. Data on clinical presentations, genetic findings, diagnostic methodologies, and outcomes were extracted and analyzed. Results: The combination of our cases and the reported cases with 22q11.2 microduplication syndrome revealed a broad phenotypic spectrum. Common clinical features include neurodevelopmental disorders, and cardiac anomalies. Importantly, the syndrome exhibits variable expressivity and reduced penetrance, with more than 70% of the findings to be inherited by one of the parents. Conclusions: 22q11.2 microduplication syndrome presents a heterogeneous clinical picture with variable expressivity and incomplete penetrance, posing challenges in diagnosis and genetic counseling, particularly when predicting prenatal outcomes. Awareness of its diverse manifestations is crucial for clinicians to consider this syndrome in the differential diagnosis and to provide informed counseling.

## 1. Introduction

Copy number variations (CNVs) constitute an important source of structural genomic variation and are increasingly recognized as major contributors to human disease, particularly in individuals with congenital anomalies and neurodevelopmental disorders. Recurrent pathogenic CNVs have been increasingly associated with developmental delay, intellectual disability, autism spectrum disorders, and multisystem congenital phenotypes, highlighting their clinical relevance in both prenatal and postnatal diagnostic settings. The widespread implementation of chromosomal microarray analysis (CMA), has significantly improved the detection rate of submicroscopic chromosomal imbalances, leading to the recognition of previously underdiagnosed genomic syndromes and expanding the spectrum of clinically relevant copy number alterations. [1,2,3].

Among recurrent CNV loci, chromosome 22q11.2 represents one of the most unstable regions of the human genome due to the presence of multiple low-copy repeats (LCR22s), which mediate non-allelic homologous recombination (NAHR) during meiosis. These recombination events generate recurrent deletions and reciprocal duplications with relatively conserved breakpoints, most commonly involving the LCR22A–D interval, although smaller nested rearrangements have also been described. The genomic architecture of this region underlies a spectrum of rearrangement-associated conditions collectively referred to as 22q11.2 rearrangement syndromes, characterized by marked phenotypic variability and diverse clinical outcomes [4,5,6,7].

While the 22q11.2 deletion syndrome, also known as DiGeorge (OMIM#188400) or velocardiofacial syndrome (OMIM#192430), represents one of the most extensively studied microdeletion syndromes with a well-established clinical spectrum, its reciprocal duplication has historically received considerably less attention [4,5]. In the typical symptoms of DiGeorge syndrome velopharyngeal insufficiency, mild facial dysmorphism, submucous cleft palate, conotruncal heart defects, and learning disabilities are included [8]. On the other hand, 22q11.2 microduplication syndrome was first described in the early 2000s following the widespread application of molecular cytogenetic techniques, revealing individuals carrying duplications of the same genomic interval associated with the deletion phenotype [6,9]. Subsequent reports demonstrated that, although sharing identical genomic breakpoints, the duplication presents with a markedly broader and often milder clinical spectrum, contributing to under-recognition and diagnostic uncertainty [10,11,12]. As a result, the clinical significance and prognostic implications of 22q11.2 duplications remain less clearly defined compared with the deletion counterpart.

The clinical phenotype associated with 22q11.2 microduplication syndrome is highly heterogeneous, ranging from apparently unaffected individuals to patients presenting with developmental delay, intellectual disability, speech and language impairment, hypotonia, and variable dysmorphic features [13,14]. Although less well characterized, duplications in the 22q11.2 region are ~2.5 times more common than 22q11.2 deletions and confer increased likelihood of autism as well as Attention-Deficit/Hyperactivity Disorder (ADHD) and cognitive impairment [15,16]. Recent research has identified intriguing gene-dosage effects of the 22q11.2 locus on brain structure, clinical symptoms, speech-language and cognition [17,18]. Congenital anomalies, including cardiac defects, palatal abnormalities, and genitourinary malformations, have also been reported, although with lower and inconsistent frequencies compared with the respective deletion syndrome. In addition, behavioral difficulties and neuropsychiatric manifestations, such as autism spectrum traits and attention-deficit disorders, have been increasingly recognized in affected individuals [18,19]. The absence of a consistent clinical presentation, together with the wide variability in severity, has complicated clinical recognition and contributed to the expanding phenotypic spectrum described across published case reports and series [20].

As shown above, the remarkable phenotypic variability associated with 22q11.2 microduplication syndrome results in significant challenges in clinical interpretation and genetic counseling. Nevertheless, during the last years, the increasing use of chromosomal microarray analysis in both prenatal and postnatal diagnostic settings, identified duplications of the 22q11.2 region with a growing frequency, often in individuals lacking a recognizable syndromic presentation [21]. Particularly, in the prenatal setting, detection of the syndrome occurs incidentally after nonspecific referral indications such as ultrasound findings or abnormal screening results, making prediction of postnatal outcome even more difficult. The absence of reliable genotype–phenotype correlations and the wide range of reported clinical outcomes complicate risk assessment and counseling, emphasizing the need for improved characterization of clinical presentations associated with this genomic rearrangement [22].

Given the considerable clinical variability associated with 22q11.2 microduplication syndrome and the challenges it poses for clinical interpretation, further characterization of its phenotypic spectrum remains essential. The aim of the present study is to contribute additional insight into the clinical manifestations and diagnostic implications of duplications involving the 22q11.2 chromosomal region. To this end, a cohort of prenatally and postnatally diagnosed cases identified at a single center through chromosomal microarray analysis was evaluated and combined with a retrospective review of previously reported cases in the literature. By integrating newly identified patients with published data, this study seeks to explore recurring clinical patterns and referral indications and to provide clinically relevant information that may support genetic counseling in both prenatal and postnatal settings.

## 2. Materials and Methods

A literature review was performed to identify published cases of 22q11.2 microduplication syndrome. Relevant studies were retrieved through searches of the PubMed database using combinations of the terms “22q11.2 duplication”, “22q11.2 microduplication”, and “22q11 duplication syndrome”. Studies published up to April 2025 were included if they reported patients with molecularly confirmed 22q11.2 duplications and provided clinical and genetic data. Studies were considered eligible if they reported patients with molecularly confirmed chromosome 22q11.2 microduplications and included sufficient clinical and/or molecular information for data extraction. Review articles, editorials, conference abstracts, studies describing only chromosome 22q11.2 deletion syndrome, and publications lacking extractable clinical data were excluded from the quantitative analysis. Review studies examined carefully to avoid duplicate entries of cases. A total of 47 studies met the inclusion criteria and were included in the final analysis. Clinical and genetic information from the eligible publications were systematically collected, including number of reported cases, indication for genetic testing, prenatal findings, clinical manifestations, and inheritance data when available. Extracted data were organized into a structured dataset to allow comparison between newly identified cases and previously reported patients (Table 1).

In parallel, chromosomal microarray analysis (CMA) performed in our laboratory, during routine examination, identified a total of 12 cases, including both prenatal and postnatal diagnoses, that carried a 22q11.2 chromosomal duplication and therefore were included in this study (Table 2). Available clinical information was collected from medical records, including referral indications, prenatal ultrasound findings when applicable, and postnatal clinical characteristics. As abnormal ultrasound findings considered structural anomalies such as cardiac malformations, increased nuchal translucency, and growth restriction, while as abnormal biochemical screening considered any deviation of the measurements of PAPP-A and b-HCG. Genetic testing was performed as part of routine clinical diagnostic evaluation using chromosomal microarray analysis (CMA). Copy number variations were detected using the Affymetrix Cytogenetics Whole-Genome CytoScan 750K array platform. The results were analyzed using the Chromosome Analysis Suite Software (ChAS ver3.1 Affymetrix, Thermo Fisher Scientific, Waltham, MA, USA) according to human genome assembly GRCh37:Feb.2009 hg19.

Detected duplications involving chromosome 22q11.2 from both our cases and the literature review, were evaluated according to genomic location, duplication size, and gene content. Descriptive analysis was performed to summarize the frequency and distribution of clinical features and referral indications within the combined cohort.

Cases included in this study were identified retrospectively through routine diagnostic genetic testing performed at our laboratory. Clinical and molecular information was analyzed in a fully anonymized manner, and no additional procedures or interventions were performed for research purposes. All patients or their legal guardians provided written informed consent for genetic testing and for the processing of their clinical and genetic information in accordance with the General Data Protection Regulation (EU 2016/679, GDPR). The study was conducted in accordance with the ethical principles of the Declaration of Helsinki. Due to the retrospective nature of the study and the use of fully de-identified data generated during routine clinical practice, formal institutional review board approval was not required.

## 3. Results

### 3.1. Present Cohort

**Figure 1:**
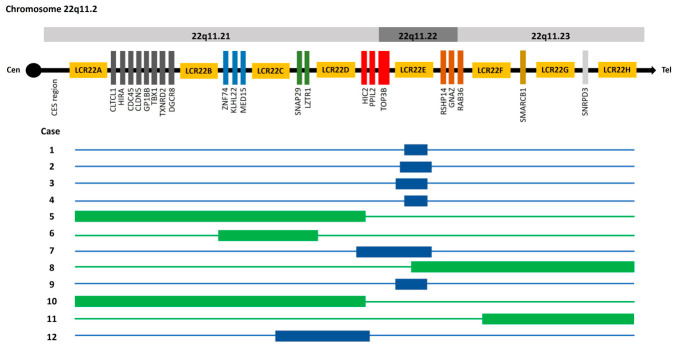
Schematic representation of the 22q11.2 duplication findings in the 12 cases from the present study. Prenatal cases are marked in green, while postnatal cases are marked in blue. Thick bars represent the duplicated genomic regions, whereas thin lines indicate the chromosomal backbone of chromosome 22q11.2.

Twelve individuals carrying duplications involving the 22q11.2 chromosomal region were identified at our center, including both prenatal and postnatal diagnoses. Among the 12 cases, 5 (41.7%) were detected prenatally and 7 (58.3%) postnatally. 10 cases were males and 2 were females (1 prenatal and 1 postnatal). All duplications were detected using chromosomal microarray analysis (array-CGH). The duplicated segments varied in size, ranging from 400 Kbp to 3152 Kbp, with differences in breakpoint localization within the 22q11.2 region (Figure 1). Only 4 (3 prenatal and 1 postnatal) out of the 12 new cases were detected between breakpoints LCR22A and LCR22D, where the critical genes of the syndrome are located.

22q11.2 microduplication syndrome is known to be associated with a broad clinical variable phenotype. Prenatal referrals were mainly due to ultrasound anomalies, (mainly increased NT), (3/5, 60%) or increased risk for trisomies identified during first-trimester biochemical screening (2/5, 40%). Postnatal referrals included dysmorphic facial features (not described), cardiac anomalies (such as Tetralogy of Fallot), and other findings (hypotonia, endocrine findings-GH deficiency, hypothyroidism). Regarding the inheritance pattern, there is no data concerning the cases of the present study.

The 12 cases were localized as follows: 3/5 prenatal cases involved the proximal region (LCR22A-D) while 2/5 the distal one (LCR22E-H). Out of the 7 postnatal cases, only one involved the proximal region, while the remaining 6 were localized between breakpoints LCR22D and LCR22E. No clear genotype–phenotype correlation was observed in our cohort with respect to duplication size or breakpoint location. This observation aligns with previous studies, which have consistently reported the absence of robust correlations between genomic architecture and clinical presentation in 22q11.2 microduplication carriers, despite attempts to stratify cases based on LCR regions or duplication size [26,37,62]. In both proximal and distal cases (of this cohort and literature), most common clinical characteristics were neurodevelopmental delay and heart defects, a finding that underlines breakpoints included did not affect the phenotype. The literature-provided data were not clear regarding whether they referred to proximal or distal cases, or whether a distinct phenotype existed between them; therefore, extracting information on genotype–phenotype correlation was difficult.

### 3.2. Literature Review

A literature review was conducted to identify previously reported cases of 22q11.2 microduplication syndrome. A total of 47 publications met the inclusion criteria and were included in the analysis.

These studies collectively described 679 individuals carrying duplications involving the 22q11.2 chromosomal region. The reported cohorts included prenatal, postnatal, and mixed case series, reflecting the increasing detection of this copy number variant both in clinical diagnostic settings and during prenatal investigations. The methods used for the detection of the duplication varied across studies. Earlier reports frequently relied on conventional cytogenetic techniques and fluorescence in situ hybridization (FISH), whereas more recent studies predominantly employed chromosomal microarray analysis (CMA). In a smaller number of cases, duplications were identified through next-generation sequencing approaches, including whole-exome sequencing (WES).

The size and genomic coordinates of the duplications varied substantially among reported individuals (120 Kbp to 6 Mbp), ranging from smaller nested duplications within the commonly rearranged region to larger segments encompassing multiple low-copy repeat (LCR) blocks of chromosome 22q11.2. Inheritance data were available in 341 cases, revealing that a considerable proportion of duplications were inherited from a parent (249/341 of cases, 73.0%), while de novo events were less frequently documented (92/341 of cases, 27.0%). However, in many reports the inheritance status was not specified (338/679 of cases, 49.8%).

The clinical characteristics reported in the literature demonstrated considerable phenotypic variability, which prompted the categorization of the observed manifestations into major phenotypic domains in the combined analysis. More specifically, neurodevelopmental manifestations represented the largest phenotypic category and included developmental delay, intellectual disability, speech and language impairment, autism spectrum disorder (ASD), attention-deficit/hyperactivity disorder (ADHD), and hypotonia. Cardiac anomalies mainly consisted of conotruncal defects, including Tetralogy of Fallot, whereas ophthalmological findings most commonly involved refractive errors. Additional systemic manifestations included renal abnormalities, endocrine disorders, hearing loss, and variable dysmorphic facial features, further illustrating the multisystemic nature and marked clinical heterogeneity of chromosome 22q11.2 microduplication syndrome. Despite the increasing number of reported cases, attempts to establish genotype–phenotype correlations have yielded inconsistent results. Several studies have explored the potential impact of duplication size and breakpoint localization on clinical outcomes; however, no reproducible patterns have been identified, reinforcing observed phenotypic variability and reduced penetrance associated with this genomic rearrangement [26,57,61].

### 3.3. Phenotypic Spectrum (Combined Analysis)

The integration of the present cohort with previously published reports allowed the evaluation of a large, aggregated dataset of individuals carrying duplications involving the 22q11.2 chromosomal region. Overall, the combined dataset included 691 individuals, comprising 679 cases from the literature (Table 1) and 12 newly identified cases from our laboratory (Table 2). To facilitate interpretation of the clinical data, cases were broadly categorized according to the timing of diagnosis into prenatal and postnatal presentations. This distinction allowed the identification of the most common indications leading to genetic testing as well as the spectrum of clinical manifestations reported after birth.

Among prenatally diagnosed individuals, the most frequent indication for genetic investigation was the presence of abnormal ultrasound findings, although abnormal biochemical screening results and other clinical indications were also reported. The spectrum of prenatal abnormalities described in the literature included cardiac anomalies, abnormal biochemical screening and other screening referral reasons. A summary of the most commonly reported prenatal findings associated with 22q11.2 microduplication is presented in Table 3.

**Figure 2:**
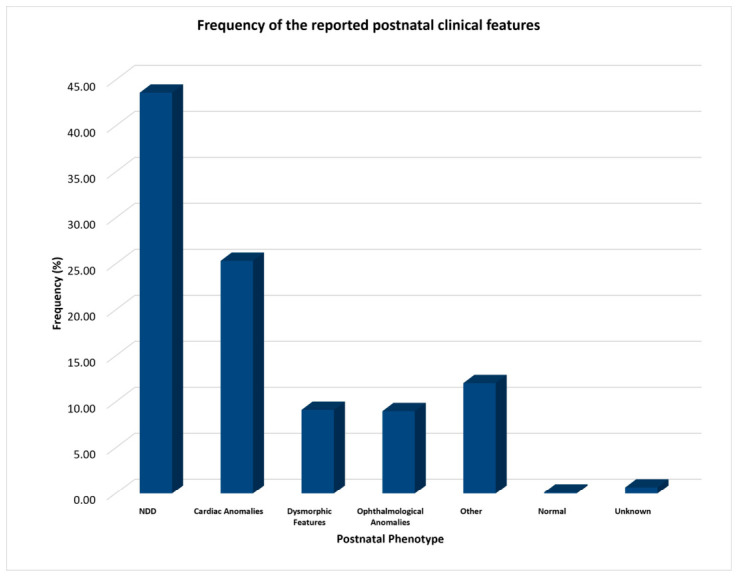
Frequency of the reported postnatal clinical features in individuals carrying a 22q11.2 microduplication based on the combined analysis of cases reported in the literature and the present cohort. NDD: Neurodevelopmental disorders.

In contrast, postnatally diagnosed individuals were most frequently referred for genetic evaluation due to neurodevelopmental disorders, cardiac anomalies, dysmorphic facial features and ophthalmological anomalies. Additional manifestations described across studies included renal anomalies, endocrine abnormalities and hearing loss, although the reported frequencies varied considerably between cohorts. The overall postnatal clinical spectrum appeared broad, involving multiple organ systems and demonstrating substantial inter-individual variability. The distribution of the most frequently reported postnatal phenotypic characteristics is summarized in Table 4, while their relative frequencies are illustrated in Figure 2.

The combined analysis also allowed the evaluation of inheritance patterns, although this information was not consistently available across all studies. When parental testing was performed, a significant proportion of duplications were reported as inherited, whereas de novo events were less commonly documented. In many cases, however, inheritance status remained unknown, primarily due to the absence of parental testing. A summary of the reported inheritance patterns across the combined dataset is presented in Table 5. When available, inheritance patterns were further examined according to duplication size and genomic location. The analysis did not reveal significant differences between the inheritance pattern and the size of the duplication, though the data were limited and maybe this finding is not necessarily valid.

Finally, the genomic characteristics of the duplications demonstrated considerable variability with respect to duplication size and breakpoint localization within the 22q11.2 region. While most duplications involved segments located between the commonly rearranged low-copy repeat regions of chromosome 22q11.2, both smaller nested duplications and larger rearrangements encompassing extended genomic intervals were reported. These findings further highlight the genomic heterogeneity associated with duplications in this region.

Where sufficient genomic resolution was available, duplications were stratified into major LCR-defined categories, including LCR22A–D (proximal/classical region), and distal or atypical (LCR22D-H) rearrangements extending beyond the canonical DiGeorge critical region. However, a substantial proportion (30.5%) of published cases could not be reliably classified due to incomplete breakpoint definition in the original reports (Table 6). This distribution likely reflects reporting bias toward clinically recognizable proximal duplications rather than true population prevalence.

## 4. Discussion

In the present study, we performed a comprehensive analysis of individuals carrying 22q11.2 duplications by combining newly identified cases from our laboratory with previously published reports. The combined dataset included a total of 691 individuals, comprising 679 cases from the literature and 12 newly identified cases, providing an updated overview of the clinical and genomic characteristics associated with 22q11.2 microduplication syndrome. To our knowledge, this represents one of the most comprehensive summaries of 22q11.2 microduplication cases reported to date. Compared with previous review articles, the present study integrates a substantially larger number of published patients together with 12 newly identified cases, providing an updated overview of the currently available evidence. Our findings further support the notion that, despite increasing recognition, the duplication counterpart remains less well characterized compared to the deletion syndrome, with limited large-scale analyses available in the recent literature [19]. The two conditions involve the same chromosomal region and may share certain features, such as neurodevelopmental disorders and congenital anomalies, but the 22q11.2 deletion syndrome is typically associated with a more consistent and severe phenotype, including well-recognized features such as heart defects and immunodeficiency. In contrast, the duplication syndrome is characterized by milder manifestations, reduced penetrance, and a higher frequency of apparently unaffected carriers, further complicating clinical interpretation and genetic counseling [8]. In addition, there is no clear evidence about which genes are critical for the 22q11.2 microduplication syndrome to be identified, while the presence of *TBX1* (OMIM#602054) is necessary for the diagnosis of 22q11.2 deletion syndrome.

One of the most consistent observations across published studies and the present cohort is the remarkable phenotypic heterogeneity associated with 22q11.2 duplications. In our combined analysis, neurodevelopmental disorders (like developmental delay, ASD, hypotonia) emerged as the most common clinical finding, reinforcing the central role of the 22q11.2 region in neurodevelopmental pathways. However, the broad distribution of additional features across multiple organ systems (such as bladder exstrophy, hearing loss, epispadias) further supports the concept that this genomic rearrangement exerts pleiotropic effects in different biological pathways [25,53]. These findings reinforce the conclusions of recent large cohort studies, such as that of Bhattarai et al. [26], which demonstrated a broad clinical spectrum ranging from asymptomatic individuals to patients with multisystem involvement, including neurodevelopmental impairment and congenital anomalies.

Similarly, recent population-based analyses of 22q11.2 copy number variants have highlighted the significant impact of these rearrangements on neurodevelopmental and cognitive traits, further supporting the central role of gene dosage effects (deletion/duplication/triplication, etc., of the chromosomal region) in shaping the phenotype [64]. The presence of apparently unaffected or mildly affected individuals within the reported cohorts further underscores the variability in clinical expressivity and contributes to the diagnostic complexity of the syndrome.

Even further, the lack of a clear genotype–phenotype correlation in 22q11.2 microduplication syndrome suggests that simple gene dosage alone is insufficient to fully explain the observed clinical variability. Increasing evidence indicates that additional factors, including the broader genomic background, epigenetic regulation, and possible other, may modulate phenotypic expression. This multifactorial model may play a role in the presence of unaffected carriers as well as in the wide spectrum of clinical manifestations observed even among individuals with similar duplications [15,17,64].

In the field of prenatal diagnosis, there are no ultrasound findings strongly associated with the duplication. However, the increasing use of chromosomal microarray analysis in prenatal procedure has led to a growing number of prenatal detections of 22q11.2 duplications. In the present study, abnormal ultrasound findings represented the most common indication for prenatal testing (46.4%), followed by abnormal biochemical screening results and other screening indications (such as family history for chromosomal abnormalities or advanced maternal age). These findings are aligned with recent prenatal series, including a recent study which reported that 22q11.2 duplications are frequently identified following nonspecific prenatal findings or as incidental results during genetic testing [22]. The absence of a specific prenatal phenotype and the wide variability in postnatal outcomes complicate clinical interpretation and make genetic counseling extremely challenging. Recent studies emphasize the complexity of this phenotypic heterogeneity among carriers and the difficulty of predicting the postnatal phenotype based on prenatal findings [26]. This uncertainty highlights a key challenge in prenatal genetics, where the detection of such variants often precedes the ability to accurately predict clinical outcomes. As a result, counseling must rely on probabilistic rather than deterministic models, taking into consideration the known variability and incomplete penetrance associated with this condition. The presence of apparently unaffected carriers further complicates clinical interpretation, as the same duplication may be associated with markedly different clinical outcomes even within the same family. Current evidence suggests that long-term prognosis ranges from normal development to significant neurodevelopmental impairment, emphasizing the absence of reliable predictors of clinical severity. Consequently, individualized genetic counseling remains essential, particularly in the prenatal setting where postnatal outcome cannot be accurately anticipated.

Evaluation of inheritance patterns across the combined dataset indicates that a substantial proportion of 22q11.2 duplications are inherited rather than occurring de novo. Among cases with available parental testing, inherited duplications were more frequently observed (36.7% when combining all inherited categories, including maternal, paternal, biparental, and unspecified inheritance), while de novo events accounted for a smaller proportion (13.7%). However, inheritance status remained unknown in nearly half of the cases (49.8%), reflecting the lack of systematic parental testing in many studies. These findings are consistent with previous reports suggesting that inherited duplications are common and may be present in apparently unaffected parents, further supporting the concept of variable expressivity and complicating genotype–phenotype correlations [19,26].

The genomic variability observed in duplications involving the 22q11.2 region is considered as one of the main reasons for the variability observed among affected individuals [19]. The presence of multiple low-copy repeat sequences (LCR22s) in this region leads to recurrent rearrangements through NAHR, resulting in duplications of varying size and breakpoint localization. In both the present cohort and the literature, duplications ranged from small nested segments to larger rearrangements encompassing multiple LCR blocks [33]. Stratification of duplications according to LCR-defined genomic architecture suggests that most clinically characterized cases involve the proximal LCR22A–D region (57.2% of cases) (Table 6). Nevertheless, the lack of uniform breakpoint reporting across published studies limits the ability to perform a fully systematic genotype–phenotype correlation across all genomic subgroups. This genomic heterogeneity has been consistently reported in recent studies and is considered a key factor underlying the broad clinical variability observed among affected individuals. Moreover, emerging data suggest that differences in duplication size and gene content may influence specific phenotypic traits, although clear genotype–phenotype correlations remain difficult to establish.

Several limitations should be considered when interpreting the findings of the present study. First, the available data were derived from previously published reports, which often differ in the level of clinical detail and phenotypic characterization provided. In addition, detailed phenotypic characterization or genotype-phenotype stratification based on LCR-defined regions and individual clinical features was not consistently available for all the cases included in our cohort, which limited the ability to perform a more refined genotype–phenotype correlation and subgroup analysis. Furthermore, detailed referral information and phenotypic descriptions were not available for all retrospectively identified cases from our institutional cohort, particularly for some of the earlier cases; therefore, these data are reported as “not available” where appropriate. Finally, the literature-derived data were subject to reporting bias and variability in the level of clinical detail provided across published studies, which may have influenced the reported frequencies of individual phenotypic features. Future large-scale curated datasets (including resources such as DECIPHER) will be essential to address this question more comprehensively. In addition, incomplete reporting of inheritance status and clinical features in a substantial proportion of cases may limit the accuracy of the combined analysis. Furthermore, the analysis of inheritance patterns in relation to duplication size and genomic location was limited by incomplete breakpoint resolution and the absence of systematic parental testing across many published reports, which precluded a fully robust stratified analysis. Additionally, potential publication bias toward clinically affected individuals should be considered when interpreting the observed phenotypic spectrum. The retrospective nature of the study and the heterogeneity of included cohorts further contribute to potential bias. Nevertheless, by integrating the largest to our knowledge number of reported cases with newly identified patients, the present study provides a comprehensive and updated overview of the clinical and genomic spectrum associated with 22q11.2 duplications.

## 5. Conclusions

In conclusion, 22q11.2 microduplication syndrome is associated with a broad and highly variable clinical spectrum involving multiple organ systems. By combining newly identified cases from our laboratory with previously reported individuals, the present study provides an updated overview of the phenotypic and genomic characteristics associated with duplications of the 22q11.2 region. This study provides an updated and comprehensive overview of the 22q11.2 microduplication cases reported to date. The findings highlight the importance of considering this genomic alteration in both prenatal and postnatal diagnostic settings. Continued accumulation of well-characterized cases will be essential for improving genotype–phenotype correlations and for supporting more accurate genetic counseling for affected individuals and their families.

## Figures and Tables

**Table 1:** Cases recorded in the literature and used in the present study.

Study	Cases Recorded	Prenatal	Postnatal
Arican et al., 2021 [23]	1	-	1
Bahji and Khalid-Khan, 2018 [24]	1	-	1
Bartik et al., 2022 [20]	122	-	122
Beaman et al., 2019 [25]	3	-	3
Bhattarai et al., 2023 [26]	216	12	204
Butensky et al., 2021 [27]	85	-	85
Chang et al., 2015 [28]	1	-	1
Chen et al., 2014 [29]	1	-	1
Clarke et al., 2009 [30]	1	-	1
Clements et al., 2017 [31]	1	-	1
Cordovez et al., 2014 [32]	2	-	2
Dale et al., 2017 [33]	1	-	1
Di Matteo et al., 2018 [34]	2	-	2
Draaken et al., 2010 [35]	2	-	2
Drmic et al., 2022 [36]	10	-	10
Dupont et al., 2015 [37]	24	24	-
Ensenauer et al., 2003 [9]	10	-	10
Evangelidou et al., 2020 [38]	1	-	1
Fischer and Klopocki, 2021 [39]	1	-	1
Forbes et al., 2016 [40]	19	-	19
Jiang et al., 2024 [22]	31	31	-
Khosroshahi et al., 2016 [41]	1	-	1
Li et al., 2020 [42]	5	5	-
Li et al., 2023 [43]	2	2	-
Mary et al., 2022 [44]	6	6	-
Moein et al., 2019 [45]	1	-	1
Pebrel-Richard et al., 2012 [46]	1	-	1
Pinchefski et al., 2017 [47]	1	-	1
Portnoi et al., 2005 [48]	2	-	2
Ribeiro-Bicudo et al., 2013 [49]	1	-	1
Schramm et al., 2011 [50]	1	-	1
Tarsitano et al., 2014 [51]	1	-	1
Turbiville et al., 2017 [52]	1	-	1
Valencia-Pena et al., 2020 [53]	1	-	1
Valvo et al., 2012 [54]	1	-	1
Vaz et al., 2015 [55]	2	-	2
Verbesselt et al., 2022 [16]	28	-	28
Verbesselt et al., 2022 [56]	19	-	19
Verbesselt et al., 2023 [57]	29	-	29
Vyas et al., 2019 [58]	1	-	1
Wang et al., 2014 [59]	1	-	1
Wentzel et al., 2008 [10]	2	-	2
Woestelandt et al., 2018 [60]	1	-	1
Woodward et al., 2019 [61]	9	-	9
Xue et al., 2021 [62]	16	15	1
Yu et al., 2008 [63]	2	-	2
Yu et al., 2019 [14]	9	-	9
Total	679	95	584

**Table 2:** Clinical and genomic characteristics of the 12 newly reported patients with chromosome 22q11.2 microduplication syndrome.

Case ID	Type (Prenatal/ Postnatal)	Genomic Coordinates (GRCh37/hg19)	Duplication Size	Referral Reason
1	Postnatal	22901371–23301460	400 Kbp	Not available
2	Postnatal	22817623–23314735	497 Kbp	Not available
3	Postnatal	22781310–23258939	477 Kbp	Not available
4	Postnatal	22901371–23301460	400 Kbp	Not available
5	Prenatal	18916961–21800471	2884 Kbp	Abnormal ultrasound findings (not described)
6	Prenatal	20730143–21464764	735 Kbp	Abnormal biochemical screening (1st trimester)
7	Postnatal	21804596–23258939	1454 Kbp	Cardiac anomalies (Tetralogy of Fallot)
8	Prenatal	22997928–25013739	2016 Kbp	Abnormal ultrasound findings (Increased NT)
9	Postnatal	22781309–23258939	478 Kbp	Dysmorphic features, Other (Hypotonia, Renal agenesis)
10	Prenatal	18648855–21800471	3152 Kbp	Abnormal biochemical screening (1st trimester)
11	Prenatal	23652548–25002659	1350 Kbp	Abnormal ultrasound findings (Increased NT)
12	Postnatal	21059669–21800471	741 Kbp	Other (GH deficiency, Familial hypothyroidism)

**Table 3:** Prenatal findings associated with 22q11.2 microduplication.

Prenatal Finding	Number of Reported Cases	Percentage (%)
Abnormal ultrasound findings	52	46.4
Abnormal biochemical screening	23	20.5
Cardiac anomalies	4	3.6
NIPT	4	3.6
Screening (Advanced maternal age, family history)	28	25.0
Other findings	1	0.9

**Table 4:** Postnatal phenotypic characteristics of individuals with 22q11.2 microduplication.

Phenotypic Feature	Individuals per Feature	Percentage (%)
Neurodevelopmental disorders	315	43.6
Cardiac Anomalies	183	25.4
Dysmorphic Facial Features	66	9.1
Ophthalmological Anomalies	65	9.0
Other	87	12.1
Normal	1	0.1
Unknown	5	0.7

**Table 5:** Inheritance patterns of 22q11.2 duplications (defined only from cases in the literature.

Inheritance Pattern	Number of Cases	Percentage (%)
Maternal	97	14.3
Paternal	81	11.9
Maternal and Paternal	1	0.2
Inherited (not defined)	70	10.3
De novo	92	13.5
Unknown	338	49.8

**Table 6:** Distribution of duplications by LCR region.

Duplication	Number of Cases	Percentage (%)
Proximal (LCR22A-D)	395	57.2
Distal (LCR22D-H)	85	12.3
Unknown	211	30.5

## Data Availability

The original contributions presented in the study are included in the article. Further inquiries can be directed to the corresponding author.
